# Leptospirosis Pathophysiology: Into the Storm of Cytokines

**DOI:** 10.3389/fcimb.2018.00204

**Published:** 2018-06-20

**Authors:** Julie Cagliero, Sharon Y. A. M. Villanueva, Mariko Matsui

**Affiliations:** ^1^Group Immunity and Inflammation, Institut Pasteur International Network, Institut Pasteur in New Caledonia, Nouméa, New Caledonia; ^2^Department of Medical Microbiology, College of Public Health, University of the Philippines, Manila, Philippines

**Keywords:** *Leptospira*, leptospirosis, inflammatory response, cytokine storm, immunoparalysis, susceptible/resistant hosts

## Abstract

Leptospirosis is a neglected tropical zoonosis caused by pathogenic spirochetes of the genus *Leptospira*. Infected reservoir animals, typically mice and rats, are asymptomatic, carry the pathogen in their renal tubules, and shed pathogenic spirochetes in their urine, contaminating the environment. Humans are accidental hosts of pathogenic *Leptospira*. Most human infections are mild or asymptomatic. However, 10% of human leptospirosis cases develop into severe forms, including high leptospiremia, multi-organ injuries, and a dramatically increased mortality rate, which can relate to a sepsis-like phenotype. During infection, the triggering of the inflammatory response, especially through the production of cytokines, is essential for the early elimination of pathogens. However, uncontrolled cytokine production can result in a cytokine storm process, followed by a state of immunoparalysis, which can lead to sepsis and associated organ failures. In this review, the involvement of cytokine storm and subsequent immunoparalysis in the development of severe leptospirosis in susceptible hosts will be discussed. The potential contribution of major pro-inflammatory cytokines in the development of tissue lesions and systemic inflammatory response, as well as the role of anti-inflammatory cytokines in contributing to the onset of a deleterious immunosuppressive cascade will also be examined. Data from studies comparing susceptible and resistant mouse models will be included. Lastly, a concise discussion on the use of cytokines for therapeutic purposes or as biomarkers of leptospirosis severity will be provided.

## Introduction

Leptospirosis is a re-emerging neglected zoonosis, caused by pathogenic spirochete bacteria from the genus *Leptospira* and estimated to infect more than a million people with approximately 60,000 deaths annually (Costa et al., 2015a,b; Picardeau, 2015). Number of fatal cases is comparable to or even higher than some other important neglected tropical diseases such as severe dengue or visceral leishmaniasis (Picardeau, 2015). Leptospirosis occurs after direct or indirect contact with bacteria shed in the urine of reservoir animals, mostly rodents, in which infection is asymptomatic and results in chronic renal carriage (Adler and de la Pena Moctezuma, 2010). Clinical manifestations in infected humans are extremely variable, including flu-like symptoms, and spontaneously resolve in 90% of cases (Haake and Levett, 2015; Gomes-Solecki et al., 2017). However, 10% of patients develop severe forms of the disease and are designated as susceptible hosts in this review. Severe human leptospirosis is characterized by multi-organ failures and is associated with a dramatic increase in the mortality rate (Adler and de la Pena Moctezuma, 2010; Haake and Levett, 2015). Hepatic dysfunctions associated with renal failure and hemorrhages constitute Weil's syndrome, a severe form of leptospirosis (Haake and Levett, 2015). Acute kidney injury (AKI) is commonly reported as an early manifestation of acute leptospirosis and could possibly evolve to chronic kidney disease (CKD) (Correa-Rotter et al., 2014; Herath et al., 2014). Severe pulmonary hemorrhagic syndrome (SPHS) with acute respiratory distress syndrome (ARDS) can also occur and can be confused with viral pneumonitis (Trevejo et al., 1998; Haake and Levett, 2015).

Although it is still unclear why leptospirosis patients present with various clinical manifestations, both innate and adaptive immune responses to *Leptospira* infection influence the outcome of the disease. Induction of an inflammatory response due to an infection with a pathogen can initiate destructive immune mechanisms leading to host tissue damages, sepsis and death. Interestingly, the cytokine storm process was suggested to have a role in the development of severe leptospirosis, especially in humans (Reis et al., 2013; Haake and Levett, 2015). In this review, we summarized evidences supporting this hypothesis, mainly based on results from studies on hosts susceptible and resistant to lethal infection.

## Inflammatory response to *leptospira* infection

During an infection, contact with pathogens activates the innate immune system by generating an inflammatory response. Microbial Pathogen-Associated Molecular Patterns (PAMPs) will be recognized by the Pattern Recognition Receptors (PRRs) expressed at the surface of innate immune cells, mainly the Toll-like receptors (TLRs) and nucleotide-binding oligomerization domain (NOD)-like receptors (NLRs) (Akira et al., 2006). The PAMPs/PRR association triggers an inflammatory cascade by activating multiple intracellular signaling pathways, including the NF-κB and activator protein 1 (AP-1) transcription factors (Schroder and Tschopp, 2010), which in turn regulate the expression of cytokines, Prostaglandins (PGs), and Nitric Oxide (NO) (Tisoncik et al., 2012; Turner et al., 2014). PGs and NO are pro-inflammatory molecules that increase arterial dilation and vascular permeability, both being key events required for the influx of immune cells (Ricciotti and FitzGerald, 2011; Wink et al., 2011). Pro-inflammatory cytokines include interleukins (IL)-1β, IL-6, IL-12, interferons (IFNs) and tumor necrosis factors (TNFs), as well as chemokines, which act as chemoattractants to recruit leucocytes to the site of tissue damage and/or infection (Tisoncik et al., 2012; Turner et al., 2014). Interestingly, AP-1 and NF-κB also modulate the pro-inflammatory response through the induction of immunomodulatory cytokines as IL-4, IL-10, IL-13 or Transforming Growth Factor-β (TGF-β) acting in concert with cytokines inhibitors to offset the massive induction of pro-inflammatory mediators (Dinarello, 2007; Turner et al., 2014).

Data show that leptospiral PAMPs stimulate innate immunity through several PRRs to produce cytokines and activate the inflammatory cascade, as was recently reviewed by C. Werts (Werts, 2017). Mechanisms underlying the specific leptospiral PAMPs/PRR-triggered inflammation have not yet been fully elucidated. Interestingly, in contrast to other bacterial LPS (e.g., from *E. coli*) that classically activates TLR4 signaling pathway, leptospiral LPS is not recognized by TLR4, but by TLR2 in human cells, while both TLR2 and TLR4 are activated in mice (Nahori et al., 2005). Moreover, TLR4-deficient mice develop clinical signs of severe leptospirosis (Gomes-Solecki et al., 2017), which show that murine TLR4 protect mice from developing leptospirosis. This species specificity in leptospiral PAMPs sensing also suggest that hosts could trigger different inflammatory responses according to their susceptibility to leptospirosis.

## Cytokine storm in severe leptospirosis

Inflammatory cytokines and cytokine regulators interact in a complex network finely controlled to clear the pathogens without excessive inflammation-induced organ damage. Indeed, severe infectious diseases are often associated with a prolonged increase in pro-inflammatory IL-1β, TNF-α, IL-6 expression, or “cytokine storm,” causing persistent inflammation and followed by a massive and systemic production of anti-inflammatory cytokines, causing a state of “immunoparalysis” (Tisoncik et al., 2012; Zhao et al., 2015). Consequently, tissue edema impairs local organ perfusion, which can result in loss of organ function; furthermore, prolonged endothelial permeabilization can lead to pathogen invasion into the bloodstream and result in a sepsis-like syndrome (Cohen, 2002; Tisoncik et al., 2012). In fact sepsis is now defined as a life-threatening organ dysfunction caused by a dysregulated host response to an infection (Singer et al., 2016) and is referred to as a “cytokine storm-induced syndrome” (Chousterman et al., 2017).

**Figure 1:**
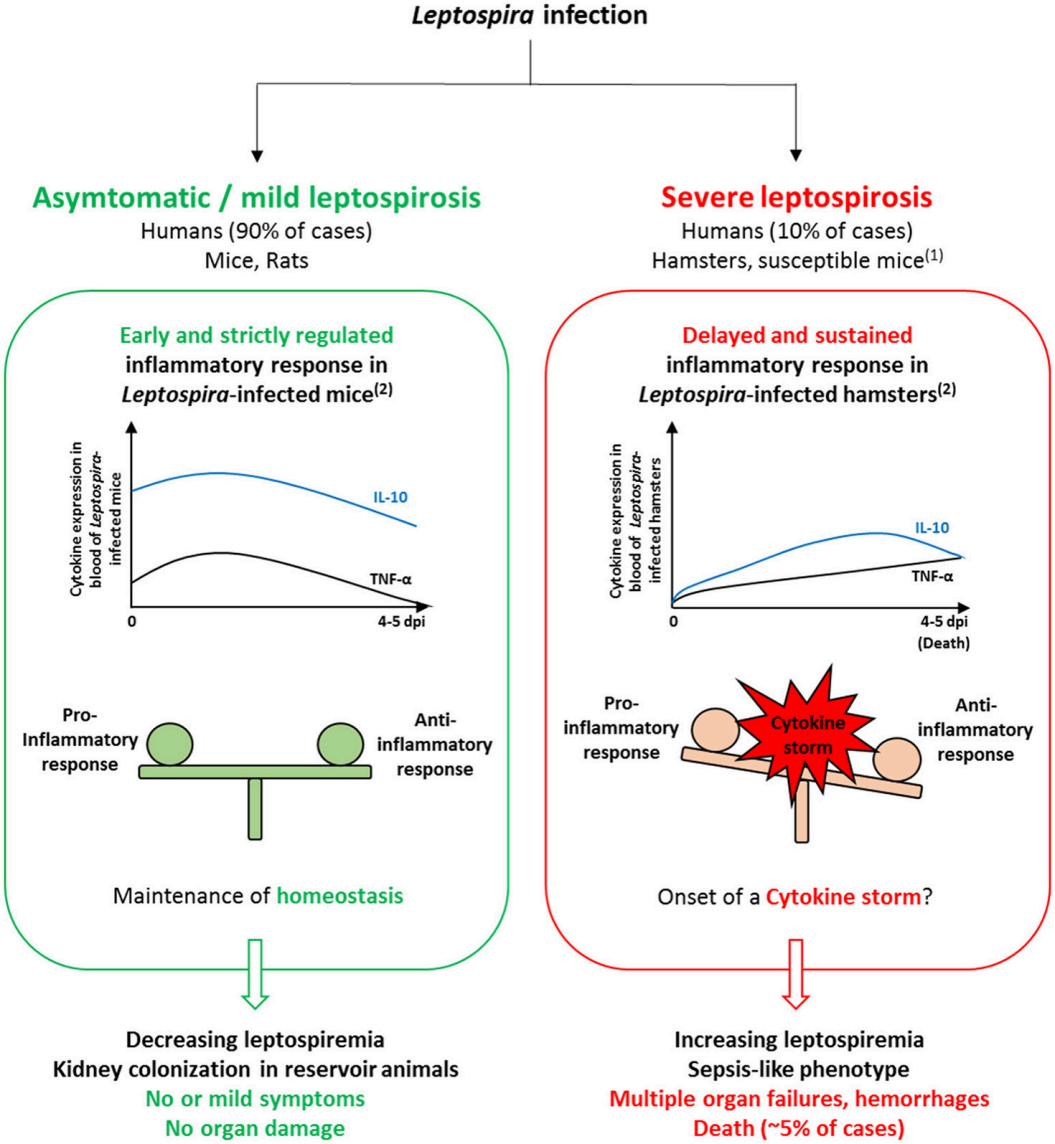
Comparison of models of *Leptospira* infection in resistant and susceptible hosts suggests the importance of the inflammatory response in influencing the disease outcome. In *Leptospira*-infected mice, the inflammatory response is rapid and strictly regulated. Consequently, homeostasis is maintained as bacteria are rapidly cleared from the blood and no or only mild symptoms develop, which relate to mild leptospirosis observed in 90% of human infections. In *Leptospira*-infected hamsters, cytokine production is delayed and sustained, which could trigger the onset of a cytokine storm and impair pathogen clearance from the blood. The resulting sepsis-like phenotype could be associated to increased bacterial load, organ failures and hemorrhages found in infected hamsters as well as in severe forms of leptospirosis in humans. ^(1)^Susceptible mouse models (e.g., C3H/HeJ strain) comprise inbred wild-type, immunosuppressed and transgenic mice, used as models for sublethal and lethal leptospirosis (see Gomes-Solecki et al., 2017 for review). ^(2)^Comparison of the inflammatory response in resistant vs. susceptible animal models is based on expression profiles found in Matsui et al. (2011). Expression levels of IL-10 and TNF-α are representative of anti- and pro-inflammatory cytokine production, respectively.

Clinical signs of severe leptospirosis in susceptible hosts include increased leptospiremia and multi-organ failure, specifically affecting kidneys, liver and lungs (Levett, 2001; Haake and Levett, 2015; Yilmaz et al., 2015). Interestingly, these clinical features meet the criterions for a sepsis diagnosis, which suggest that development of severe leptospirosis could be associated with a dysregulated inflammation. Interestingly, data obtained from clinical studies support this hypothesis. The very first quantifications of cytokines in human leptospirosis showed significant increase in TNF-α level from patient sera (Estavoyer et al., 1991; Tajiki and Salomão, 1996; Tajiki et al., 1997). Further investigations reported higher production of cytokines in severe compared to mild disease (Reis et al., 2013; Mikulski et al., 2015; Chirathaworn et al., 2016). Serum levels of pro-inflammatory IL-6, chemokine IL-8 and anti-inflammatory IL-10 were significantly higher among patients that developed SPHS compared to non-SPHS leptospirosis patients (Reis et al., 2013). In addition, concentrations of these cytokines were elevated in sera from patients with organ dysfunction compared to mild cases without organ involvement (Chirathaworn et al., 2016). Experimental infection also showed evidence of differential pattern of cytokine expression depending on resistance or susceptibility of animal models (Figure 1). Strictly regulated induction of pro-inflammatory cytokines IL-1β, IL-6, TNF-α and chemokines CXCL10/IP-10 and CCL3/MIP-1α in resistant mice contrasted with delayed and massive overexpression in susceptible hamsters developing severe tissue lesions (Matsui et al., 2011). Overexpression of anti-inflammatory IL-10 was faster and at higher levels in resistant mice than in hamsters (Matsui et al., 2017). Moreover, expression of TNF-α, IL-1α, and IL-10 were significantly higher in lethally infected hamsters compared to survivors (Vernel-Pauillac and Goarant, 2010). Based on these, dramatic imbalance in the cytokine production upon *Leptospira* infection might play a considerable role in the development of severe leptospirosis.

## Role of cytokines in the pathophysiology of severe leptospirosis

### Pro-inflammatory cytokines

Although TNF-α was the first cytokine to be associated with leptospirosis severity (Estavoyer et al., 1991; Tajiki and Salomão, 1996), its precise role in the disease pathophysiology is still under debate. Macrophage apoptosis induced by *L. interrogans in vitro* is triggered via caspase-8 and caspase-3-dependant pathways (Jin et al., 2009), and *Leptospira* were suggested to induce apoptosis in organs though the caspase-3-dependent pathway in infected mice (Marinho et al., 2015). Thus, TNF-α might play a role in *Leptospira*-induced apoptosis as it can activate caspase-3- and caspase-8-associated apoptosis (Zhao et al., 2001; Wang et al., 2008).

Kyriakidis et al. reported that TNF-α was the only cytokine found associated with SHPS in leptospirosis patients (Kyriakidis et al., 2011). Alteration of sodium channel was suggested as a cause for the pulmonary damages observed in susceptible *Leptospira*-infected hamsters (Andrade et al., 2007). Interestingly, TNF-α, together with IL-1β, regulate the expression of sodium channel sub-units *in vitro*, which could be part of pulmonary damages underlying mechanism (Yamagata et al., 2009).

Pro-inflammatory IL-6 is induced by TNF-α and IL-1β and produced by a large number of immune and non-immune cells. High concentration of IL-6 is an indicator of septic shock and correlates to leptospirosis severity and SPHS (Reis et al., 2013; Schulte et al., 2013; Papa and Kotrotsiou, 2015). As TNF-α and IL-1β, IL-6 can also activate the coagulation system, especially in endotoxemic models (Schulte et al., 2013). This could relate to clinical bleedings, especially in SHPS (Haake and Levett, 2015), and activation of coagulation (Wagenaar et al., 2010) observed in severe leptospirosis.

High levels of chemokines are found in susceptible hamsters (Matsui et al., 2016), and are associated with organ damage and poor outcome (Reis et al., 2013; Papa and Kotrotsiou, 2015). Notably, higher level of CXCL8/IL-8 expression was found in patients with severe clinical signs and was associated with mortality (Wagenaar et al., 2009a,b). Moreover, high levels of adhesion molecules, ICAM-1 or VCAM, are also associated with leptospirosis-induced organ damages (Del Carlo Bernardi et al., 2012). Chemokines along with endothelial adhesion molecules are induced by TNF-α, which promotes leukocytes attraction and extravasation into injured tissue. Their prolonged production can also promote endothelial barrier disruption and diapedesis, which could explain hemorrhages and immune cells infiltrations in tissues associated with severe leptospirosis.

Together with IL-1β, TNF-α promote the activation of immune cells as macrophages, and subsequent secretion of immuno-regulators including pro-inflammatory factors that amplify the inflammatory response. Moreover, high TNF-α and IL-1 circulating levels are considered as the hallmarks of a cytokine storm and their role in sepsis pathophysiology has been largely investigated (Schulte et al., 2013). These data place TNF-α and IL-1β as master conductors of the awry inflammatory response and the subsequent cytokine storm-induced sepsis observed in severe leptospirosis. However, it also emphasizes that these cytokines may not be the best candidates for investigating specific immune responses to a *Leptospira* infection and for selection of biomarkers for severity prognosis. Genetic studies showing no correlation between TNF-α polymorphisms and leptospirosis outcome are in good accordance with this hypothesis (Lingappa et al., 2004).

### Anti-inflammatory and immunomodulatory cytokines

Following the onset of a cytokine storm, systemic production of IL-10 is considered as a hallmark of an attempt to restore immunological homeostasis (Tisoncik et al., 2012). High level of IL-10 in septic patients significantly correlates with sepsis and death suggesting profound immunosuppression (Gogos et al., 2000). It also influences the clearance of microorganisms in the host (Duell et al., 2012). In Lyme borreliosis, a disease due to an infection by another spirochete, *Borrelia burgdorferi*, contribution of IL-10 in the attenuation of tissue lesions was shown through the modulation of pro-inflammatory cytokines (Brown et al., 1999; Gautam et al., 2011). Inhibition of endogenous IL-10 also enhanced *Borrelia* elimination (Brown et al., 1999).

In human leptospirosis, controversial results showed either higher levels of IL-10 (Reis et al., 2013; Chirathaworn et al., 2016) or no significant difference in IL-10 levels (Wagenaar et al., 2009a; Mikulski et al., 2015) between mild and severe forms. However, asymptomatic leptospirosis presented an anti-inflammatory response with higher IL-10-producing CD4+ T-cells compared to patients with severe or mild leptospirosis (Volz et al., 2015). Differences in time of sampling might explain this discrepancy. Interestingly, in a murine Cecal Ligation and Puncture (CLP)-induced sepsis model, the inhibition of IL-10 at the time of CLP worsened mortality rate whereas it increased survival when administered 12 h later (Song et al., 1999). Thus, IL-10 contribution in leptospirosis pathophysiology probably depends on the stage and severity of the disease. Altogether, these findings suggest that IL-10 could either be protective by counterbalancing pro-inflammatory cytokines during the early stage of the disease, or have harmful effect in the leptospirosis outcome acting on bacterial burden. Finally, genetic variations could also contribute to the IL-10 dual role since IL-10 promoter polymorphisms were associated with lower LPS-dependent IL-10 production and with the development of sepsis in patients with major trauma (Zeng et al., 2009).

Inflammatory response is also restricted by regulatory cytokines, as IL-4 or IL-13, promoting T helper (Th) lymphocyte differentiation toward Th2 lineage, suppressing tissue-damaging effects of sustained inflammation (Opal and Depalo, 2000). Moreover, aberrant production of these cytokines facilitates pathogen invasion during infection (Wynn, 2015). IL-4 is induced during leptospirosis with late but massive overexpression in blood from experimentally infected hamsters (Vernel-Pauillac and Merien, 2006). Serum levels of IL-4 were also increased in human patients (Reis et al., 2013) and frequencies in IL-4 and IL-4R receptor gene polymorphisms were significantly higher in leptospirosis patients compared to healthy subjects (Fialho et al., 2009). Similarly to IL-10, the role of IL-4 in sepsis is still under debate (Chousterman et al., 2017)

Thus, induction of IL-4 and IL-10 might play an important role in leptospirosis pathophysiology that still needs to be precisely described. Indeed, these cytokines could relate to impaired bacterial clearance by restricting pro-inflammatory responses and consequently triggering immunoparalysis related to fulminant septicemia in human leptospirosis (Tisoncik et al., 2012; Zhao et al., 2015).

## Resistant phenotype to *leptospira* infection: role of the immune response

Although hamsters and guinea pigs are suitable models to study acute leptospirosis, the cost of their handling and the lack of experimental tools have limited their use in laboratories. As mentioned above, mice, which are much easier to handle, are resistant to *Leptospira* infection and become chronic carrier of the bacterium. However, variable degrees of susceptibility have been observed using different mice strains, which allowed scientists to consider mice as suitable models for studying severe leptospirosis. Consequently, a large number of transgenic, mutant, or immunosuppressed mice are now being used as models of chronic, sub-lethal and lethal leptospirosis (see Gomes-Solecki et al., 2017 for review).

Contrasting with delayed and uncontrolled cytokine production observed in susceptible hosts, the inflammatory response in resistant models is characterized by an early but tightly regulated induction of pro-inflammatory cytokines, and a fast and high overexpression of the anti-inflammatory IL-10 (Figure 1). Recently, higher chemokines levels including CCL5/RANTES and CCL8/MCP-2 were quantified in organs from resistant (BALB/c) compared to susceptible (TLR4-defective C3H/HeJ) mice 24 h post-infection (Domingos et al., 2017). Moreover, high expression of chemokines CXCL1/KC, CXCL2/MIP-2, CCL5/RANTES, as well as IL-1β, TNF-α, and IL-10 was observed in sub-lethally infected C3H/HeJ mice (Richer et al., 2015; Sullivan et al., 2017). This phenotype specificity in the immune response is supported by previous data showing that expression of TNF-α and of CXCL2/MIP-2 chemokine is delayed in organs from susceptible mice (da Silva et al., 2012). Thus, time of induction of chemokines and key cytokines seems to be determinant for the development of a resistant phenotype to *Leptospira* infection.

Moreover, and supporting the possible dual role of IL-10, recent studies reported improved bacterial clearance in mouse kidneys in the absence of IL-10 compared to control animals (Devlin et al., 2017; Matsui et al., 2017). Interestingly, IL-10 deficiency did not affect *Leptospira*-dependent induction of TNF-α and IL-6 while it enhanced IL-1β and IFN-γ overexpression. Thus, IL-10 might inhibit the effective clearance of *Leptospira* through the early regulation of particular inflammatory cytokines, leading to the persistence of bacteria and allowing chronic carriage in kidneys of resistant animals. It is noteworthy that no clinical signs were observed in *Leptospira*-infected Il-10^−/−^ mice (Devlin et al., 2017) while IL-10 neutralization led to weight loss in infected OF1 mice (Matsui et al., 2017).

Finally, expression of the anti-inflammatory TGF-β is not modified in the blood (Vernel-Pauillac and Goarant, 2010; Fujita et al., 2015) while induced in the kidneys (Lowanitchapat et al., 2010) from *Leptospira*-infected hamsters during the acute stage of the disease. Interestingly, renal TGF-β1 expression is downregulated in asymptomatic mice during chronic carriage of *Leptospira* (Matsui et al., 2016) contrasting with unchanged expression level in fibrotic kidneys from susceptible mice compared to control (Fanton d'Andon et al., 2014; Ferrer et al., 2014). Consistently with the pro-fibrotic role of this cytokine in renal fibrogenesis (Higgins et al., 2018), and beyond its anti-inflammatory effect, this cytokine might specifically participate in renal pathophysiology of leptospirosis.

## Discussion

Inflammation is essential for the resolution of microbial infections and involves complex processes that finely coordinate cytokine production. Dysregulation of these mechanisms can trigger cytokine storm and related multi-organ failures as observed during severe leptospirosis and sepsis. Interestingly, the Jarisch-Herxheimer Reaction (JHR) noticed among leptospirosis patients is also characterized by large cytokine overproduction induced by massive bacterial product release consequent to antibiotic treatment (Friedland and Warrell, 1991; Guerrier and D'ortenzio, 2013; Guerrier et al., 2017). It is worth noticing that anti-TNF-α antibody therapy was proposed to prevent and ameliorate the JHR (Pound and May, 2005; Butler, 2017). However, despite the well-established involvement of cytokines in sepsis as observed in severe leptospirosis, no efficient treatment targeting inflammation was clinically validated. Indeed, data obtained from calibrated pre-clinical models can barely be translated to human diseases (van der Worp et al., 2010; McCarron et al., 2015). Thus, improving knowledge of the host innate immunity in leptospirosis is a major challenge to ameliorate therapeutic approaches, especially in severe forms of leptospirosis that are associated with a dramatic increase in mortality rate.

During *Leptospira* infection, inflammatory mediators are rapidly regulated in resistant models contrasting with awry production in susceptible hosts, and cytokines were thus proposed as promising biomarkers of the disease outcome in several clinical studies. However, larger and multi-factorial investigations are still required, including cytokine kinetics critical over the course of infection. Unfortunately, in patients, precise time of infection and infecting dose are hardly determined. Moreover, mediators are usually quantified in sera, but specific profiling of host response, including the identification of cellular sources for their production, in altered tissues would necessarily improve our understanding of inflammatory dysfunctions in organs.

Management of severe leptospirosis is also complex due to the diversity of clinical symptoms and immunological profiles found among patients. Human genetic background should be taken into consideration as highlighted by susceptible TLR4 deficient C3H/HeJ mice. However, only a few studies have investigated the correlation between genetic variability and leptospirosis severity and results are still under debate (Fialho et al., 2009; Esteves et al., 2014; Cedola et al., 2015). Finally, pathogen virulence will inevitably influence the host immune response. Several leptospiral components were described as virulence factors, including genes implicated in motility and LPS synthesis, but also genes whose functions still remain undescribed.

## Author contributions

MM drafted the review. JC, SV, and MM contributed to the writing process.

### Conflict of interest statement

The authors declare that the research was conducted in the absence of any commercial or financial relationships that could be construed as a potential conflict of interest.
